# How do living conditions affect the gut microbiota of endangered Père David’s deer (*Elaphurus davidianus*)? Initial findings from the warm temperate zone

**DOI:** 10.7717/peerj.14897

**Published:** 2023-02-24

**Authors:** Hongyu Yao, Qiying Mo, Hong Wu, Dapeng Zhao

**Affiliations:** Tianjin Key Laboratory of Conservation and Utilization of Animal Diversity, College of Life Sciences, Tianjin Normal University, Tianjin, China

**Keywords:** 16S rRNA sequencing, Fecal sample, Père David’s deer, Gut microbiota, Reintroduction

## Abstract

Reintroduction is an effective strategy in the conservation of endangered species under scientific monitoring. Intestinal flora plays an important role in the envir onmental adaptation of endangered Père David’s deer (*Elaphurus davidianus*). In this study, 34 fecal samples from *E. davidianus* were collected from different habitats in Tianjin city of China to investigate differences in the intestinal flora under captive and semi-free-ranging conditions. Based on 16S rRNA high-throughput sequencing technology, a total of 23 phyla and 518 genera were obtained. Firmicutes was dominant in all individuals. At the genus level, *UCG-005* (13.05%) and *Rikenellaceae_RC9_gut_group* (8.94%) were dominant in captive individuals, while *Psychrobacillus* (26.53%) and *Pseudomonas* (11.33%) were dominant in semi-free-ranging individuals. Alpha diversity results showed that the intestinal flora richness and diversity were significantly (*P* < 0.001) higher in captive individuals than in semi-free-ranging individuals. Beta diversity analysis also showed a significant difference (*P* = 0.001) between the two groups. In addition, some age- and sex-related genera such as *Monoglobus* were identified. In summary, the structure and diversity of intestinal flora showed significant habitat variation. This is the first time an analysis has been undertaken of the structural differences of the intestinal flora in Père David’s deer, under different habitats in the warm temperate zone, providing a reference basis for the conservation of endangered species.

## Introduction

Within conservation biology, reintroduction is a widespread technique that has helped many endangered or extinct wildlife species to recover their population size, including mammals, birds, and invertebrates (Seddon, Armstrong & Maloney, 2007; Sutton, 2015; Corlett, 2016). However, many factors affect the success rate of reintroduction, and poor performance is reported on reintroducing threatened or endangered species from captivity to the wild (Reading, Miller & Shepherdson, 2013; Seddon et al., 2014). Monitoring of released free-ranging animals is of utmost importance in improving the success rate of reintroduction (Seddon, Armstrong & Maloney, 2007; Yang et al., 2018). Previous research has shown that reintroduction has helped a number of species, such as giant pandas (Yang et al., 2018), peregrine falcons (Jacobsen et al., 2008), and Texas horned lizards (Williams, Rains & Hale, 2019) to successfully reappear and reproduce in their historic ranges. One representative success story is the reintroduction of Père David’s deer (*Elaphurus davidianus*) (Cheng et al., 2021). *E. davidianus* was endemic to China and ranged from Liaoning Province in northern China to Zhejiang Province in southern China, from 19 degrees North Latitude to 47 degrees North Latitude (Cheng et al., 2021). *E. davidianus* originated in the early Pleistocene period and reached their peak over 3,000 years ago. However, due to anthropogenic and natural pressures including human hunting, environmental destruction, climate change, and war, *E. davidianus* disappeared from their original habitats in the early 20th century (Cheng et al., 2021). The endangered species was reintroduced to China from England in 1985 and has bred in many areas since (*e.g*., Beijing, Jiangsu, Hubei, and Hunan), with a total population of nearly 10,000 to date (Cheng et al., 2021). Although the total number of *E. davidianus* in China is growing, the majority of deer live in captivity, and much work remains to be done for successful reintroduction and rewilding across their historic habitats (Sutton, 2015). Natural food selection and health of *E. davidianus* during the process of living from captivity to the wild are key aspects of reintroduction work (Sun et al., 2019).

In order to solve this problem effectively, intestinal microbiota monitoring, based on non-invasive sampling technology, can be used to detect the relationship between gut microbiome and health of the host animal, especially for threatened species. Research has shown that gut microbiota plays an essential role in contributing to food digestion and disease immunity of their host (Pan & Yu, 2014). Numerous factors can influence gut bacterial diversity, such as diet (Jiang et al., 2020), sex (Kim et al., 2020), and age (Jami et al., 2013). To date, investigations on intestinal microbiota of *E. davidianus* are still in their infancy, and only four related studies have been published (Zhang et al., 2018; Sun et al., 2019; Wang et al., 2019; Zhen et al., 2022). These studies have all focused on populations living in subtropical zones as well as those living in transition areas from warm temperate zones to subtropical zones. There is a great knowledge gap on the gut bacteria community of *E. davidianus* in the warm temperate zone, and no studies have been conducted to investigate the relationship between sex or age and gut microbiota establishment in *E. davidianus*.

The main purpose of this study was to analyze differences in gut microbiota composition and diversity of *E. davidianus* living in the warm temperate zone, under captive and semi-free-ranging living conditions, for the first time. We also analyzed the differences in gut microbiota among captive individuals of different sexes and ages. The results of this study could provide a scientific reference for the implementation of comprehensive reintroduction of *E. davidianus* in the future, as well as the scientific management and conservation of this endangered species in related protected areas.

## Materials and Methods

### Study site and sample collection

In this study, fecal samples of *E. davidianus* were obtained using a non-invasive sampling technique from different rearing environments in Tianjin city, located in North China (38°34′–40°15′N, 116°43′–118°04′E) (Wu et al., 2021). It has a semi-humid monsoonal climate with an annual average temperature of about 14 °C. We collected a total of 34 fecal samples between October and December 2021. Among them, samples from six females (NO. CF01-CF06), seven males (NO. CM01-CM07), and six juveniles (NO. CJ01-CJ06) were collected from captive groups (C group) in Tianjin Zoo. Fifteen fecal samples (NO. SF01-SF15) were obtained from semi-free-ranging groups (S group) in Tianjin Qilihai Wetland. The main diet of *E. davidianus* was provided by keepers in Tianjin Zoo (Table S1), while the semi-free-ranging group foraged for plants by themselves.

In this study, we distinguished adults and juveniles by body size, and identified the sex of adult individuals based on the existence of antlers when sampling at Tianjin Zoo. The collected fecal samples were sterilized in 5 ml tubes. The samples were stored temporarily in a refrigerated insulated box, then brought back to the laboratory for freezing at –80 °C.

### DNA extraction, amplification, and sequencing

All samples were extracted using the TIANamp Stool DNA Kit (TIANGEN, Sichuan, China). Specific primers with barcodes were synthesized according to the specified sequencing region. PCR amplification was performed according to the manufacturer’s instructions, with three replicates per sample. PCR products from the same samples were mixed and detected by 2% agarose gel electrophoresis. PCR products were recovered using AxyPrepDNA gel recovery kit (AXYGEN Corporation, Silicon Valley, CA, USA). Detection and quantification were performed by QuantiFluor-ST™ Blue Fluorescence Quantification System (Promega, Madison, WI, USA). Purified PCR amplicons were sequenced on the Illumina MiSeq platform at Shanghai Majorbio Bio-pharm Technology Co., Ltd.

### Bioinformatics, statistical analyses, and functional prediction

The raw data obtained from MiSeq sequencing were optimized using Qiime (version 1.9.1; http://qiime.org/). Sequences with at least 97% identity were subjected to Operational Taxonomic Unit (OTU) clustering analysis using Uparse (version 7.0.1090; https://drive5.com/uparse/). The taxonomic analysis of I was performed by RDP Classifier (version 2.11; http://rdp.cme.msu.edu/classifier/classifier.jsp). Rarefaction curves were created to express the species richness of each sample and the reasonableness of the sequencing depth. The Wilcoxon test was applied to detect differences in the abundance of flora between different groups at the phylum and genus level. LEfSe analysis (Score >4) was used to seek biomarkers with significant differences between the groups. In this study, Chao1, ACE, Shannon, Simpson, and Coverage alpha diversity indexes were determined using mothur software (version v.1.30.1; https://mothur.org/wiki/mothur_v.1.9.0/), demonstrating the microbial community richness and diversity in each sample. T-test analysis based on alpha diversity index was used to identify any significant differences between the groups. Principal Coordinates Analysis (PCoA) of beta diversity was implemented to visualize similarities or dissimilarities of microbial community diversity between samples. Microbial functional prediction was executed by PICRUSt based on high-quality sequences.

## Results

### Sequencing data and microbiota composition

In this study, a total of 6,922,773 optimized sequences were obtained after denoising all 34 samples by Illumina MiSeq sequencing with an average sequence length of 416 bp, ranging from 43,036 to 306,943 sequences in all samples (Table S2). By performing clustering on all sequences, a total of 3,940 OTUs with a 97% sequence similarity threshold were retrieved. The taxonomic analysis OTU showed that the gut microbiota of *E. davidianus* could be divided into 23 phyla, 48 classes, 127 orders, 241 families, and 518 genera.

### Microbiota composition and relative abundance of all samples

The rarefaction curve based on OTUs tended to gradually flatten, suggesting that the fecal samples collected in our study were enough to analyze and reflect the maximum level of bacterial diversity (Fig. S1). There were 1,799 OTUs found to be shared by all samples, while the number of OTUs shared by the C group and S group was 1,618 and 578 under the same sequencing depth, respectively (Fig. S1).

**Figure 1:**
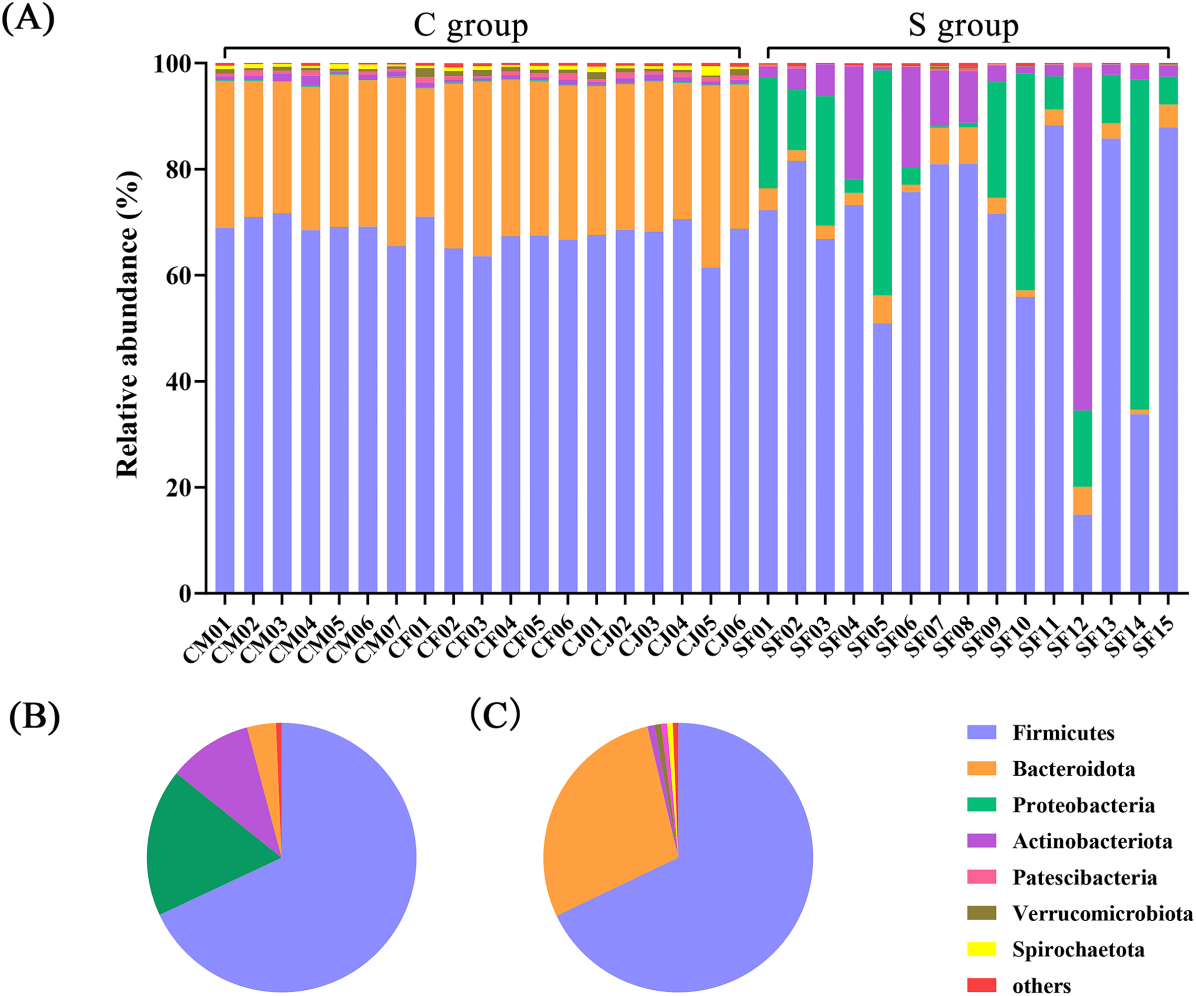
The composition of the intestinal flora from Père David’s deer at phylum level. (A) Microbial structure of all fecal samples at phylum level. The pie diagram shows the most abundant phylum in the semi-free-ranging group (B) and captive group (C).

At the phylum level, a total of 23 prokaryotic phyla were identified based on the 16S rRNA sequencing. The gut microbiota from the C group were dominated by Firmicutes (67.89%) and Bacteroidota (28.43%), followed by Actinobacteriota (0.85%) and Verrucomicrobiota (0.78%). The gut microbiota of the S group were dominated by Firmicutes (68.02%) and Proteobacteria (17.74%), followed by Actinobacteriota (10.09%) and Bacteroidota (3.47%) (Fig. 1 and Table S3).

**Figure 2:**
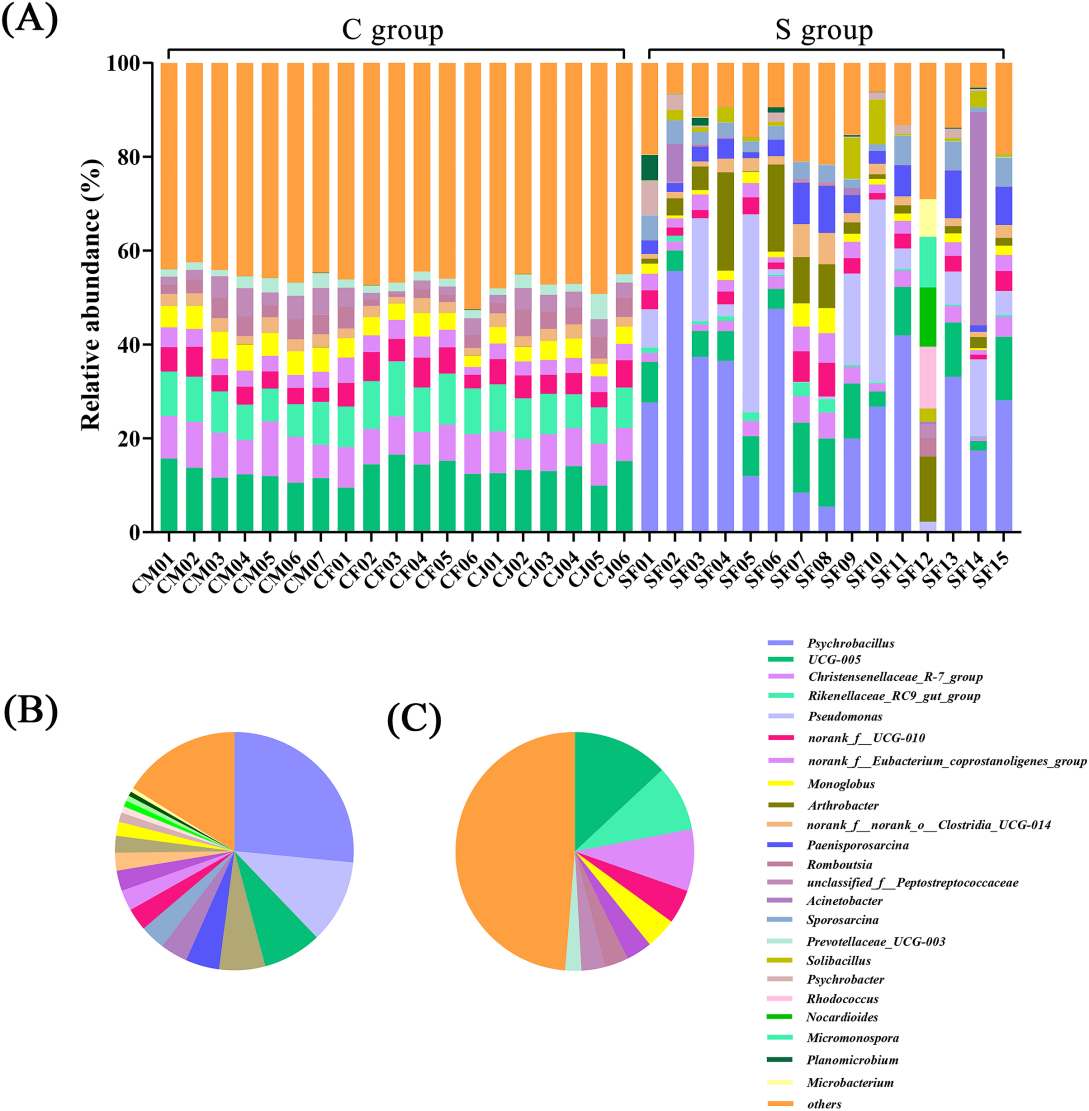
Composition of the intestinal flora from Père David’s deer at genus level. (A) Microbial structure of all fecal samples at genus level. The pie diagram shows the most abundant genus in the semi-free-ranging group (B) and captive group (C).

In terms of genus level, there were 15 genera with more than 3% abundance in all samples. The most abundant genera in the C group were *UCG-005* (13.05%), *Rikenellaceae_RC9_gut_group* (8.94%), *Christensenellaceae_R-7_group* (8.37%), *norank_f_UCG-010* (4.64%), *Monoglobus* (4.20%), *norank_f_Eubacterium_coprostanoligenes_group* (3.50%), *Bacteroides* (3.22%), and *Romboutsia* (3.20%). The most abundant genera in the S group were *Psychrobacillus* (26.53%), *Pseudomonas* (11.33%), *UCG-005* (7.98%), *Arthrobacter* (6.22%), *Paenisporosarcina* (4.58%), *Sporosarcina* (3.71%), *Acinetobacter* (3.42%), and *norank_f_UCG-010* (3.04%) (Fig. 2 and Table S4). The stacked percentage histograms of relative abundance at the phylum level (others <1%) and genus level (others <5%) were compared to visualize the relative abundance of intestinal flora in Figs. 1A and 2A, respectively.

**Figure 3:**
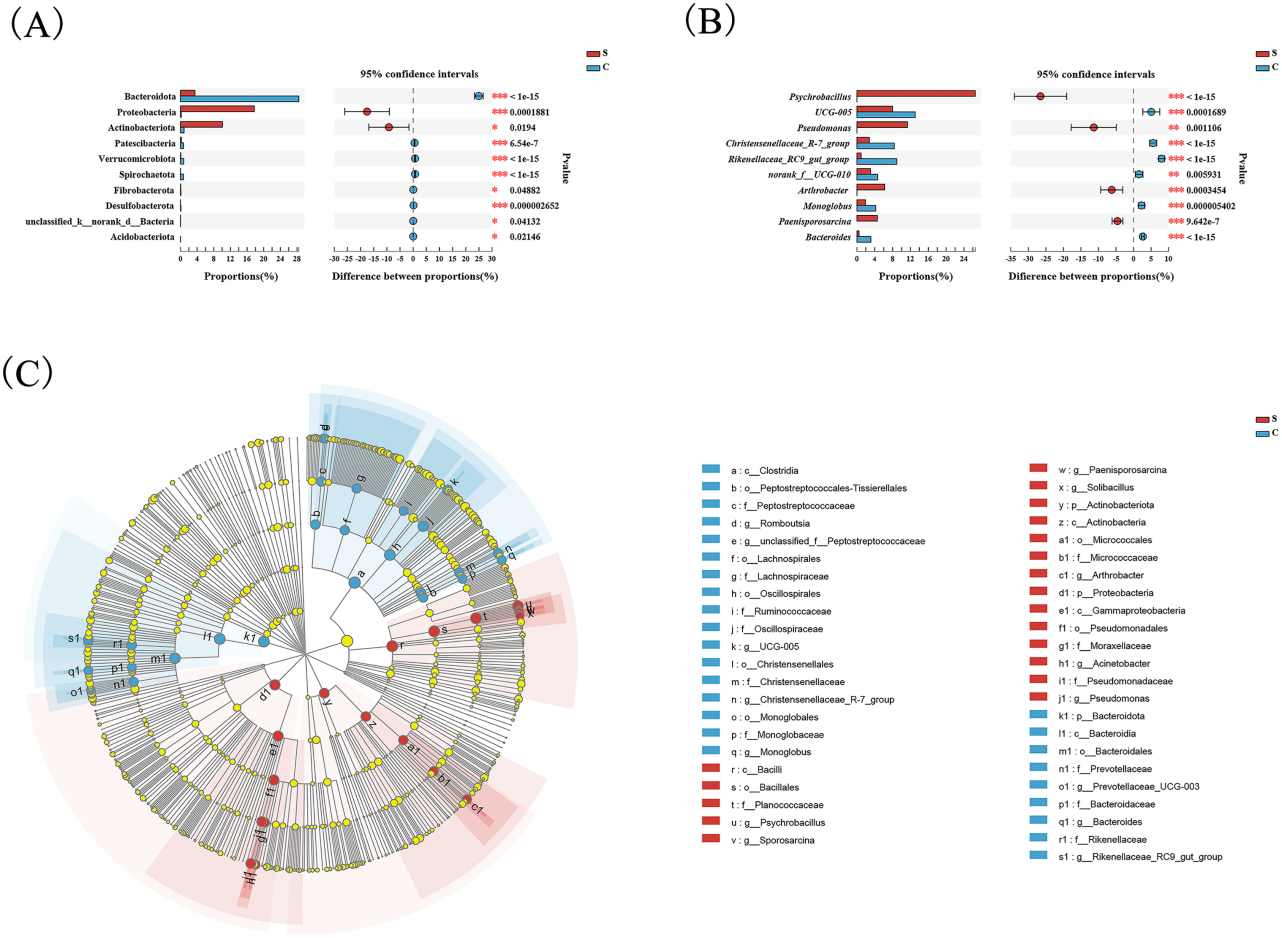
Comparison of differences in intestinal flora between semi-free-ranging and captive individuals. Differential analysis of dominant bacterial phyla (A) and genera (B) between semi-free-ranging group and captive group based on Wilcoxon tests. LEfSe analysis based on characterizing discriminative features of OTUs (C). *P < 0.05, **P < 0.01, and ***P < 0.001.

Wilcoxon tests at the phylum level showed that the percentage of Proteobacteria and Actinobacteriota in the C group were significantly lower than the S group, while the percentage of Bacteroidota was significantly higher than the S group (Fig. 3A). At the genus level, *Psychrobacter*, *Pseudomonas*, *Arthrobacter*, and *Paenisporosarcina* in the S group were significantly higher than C group, while *UCG-005*, *Christensenellaceae_R-7_group*, *Rikenellaceae_RC9_gut_group*, *norank_f_UCG-010*, *Monoglobus*, and *Bacteroides* were significantly lower than the C group (Fig. 3B).

LEfSe analysis identified 26 and 19 taxa (LDA = 4.0) with discrepancies in relative abundance in the C group and S group, respectively. The results of LEsfe analysis indicated that biomarkers in the S group were *Pseudomonas*, *Acinetobacter*, *Arthrobacter*, *Solibacillus*, *Paenisporosarcina*, *Psychrobacillus*, and *Sporosarcina*. The biomarkers in the C group were *Romboutsia*, *unclassified_f_Peptostreptococcaceae*, *UCG-005*, *Christensenellaceae_R-7_group*, *Monoglobus*, *Prevotellaceae_UCG-003*, *Bacteroides*, and *Rikenellaceae_RC9_gut_group* (Fig. 3C).

### The alpha and beta diversity of gut microbiota from different habitats

**Figure 4:**
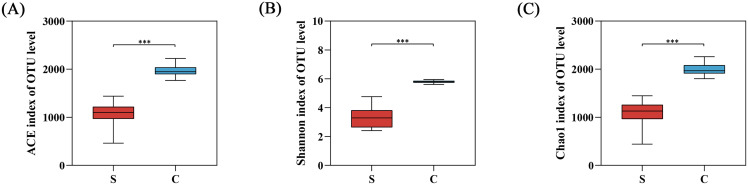
The alpha diversity between semi-free-ranging group and captive group. (A) Bacterial community richness (ACE index); (B) bacterial community diversity (Shannon index); (C) bacterial community diversity (Chao1 index). ***P < 0.001.

The alpha diversity was calculated with a T-test using mothur in our study (Table 1 and Fig. S2). The alpha diversity index showed that Chao1, ACE, and Shannon indexes of the C group were significantly higher than the S group, reflecting the richness and diversity of gut microbiota in C group (Fig. 4).

**Table 1:** Estimated OTU richness and diversity indexes for each fecal sample of Père David’s deer.

Sample ID	Shannon	ACE	Chao	Coverage
CM01	5.871300	1,936.906	1,980.016	0.990164
CM02	5.863987	1,925.843	1,964.000	0.990009
CM03	5.704975	1,766.558	1,806.000	0.991122
CM04	5.725652	1,946.295	1,967.660	0.989750
CM05	5.769444	1,992.756	2,051.708	0.989051
CM06	5.754028	1,910.045	1,908.409	0.990216
CM07	5.611283	1,923.299	1,944.030	0.989647
CF01	5.846649	1,950.132	1,966.755	0.990397
CF02	5.945778	2,223.291	2,260.692	0.987654
CF03	5.786992	1,798.043	1,801.505	0.991148
CF04	5.935381	2,057.705	2,084.868	0.989155
CF05	5.835790	2,041.680	2,045.303	0.989000
CF06	5.781073	2,000.002	2,097.222	0.988818
CN01	5.915355	1,862.732	1,884.561	0.990837
CN02	5.670464	2,111.680	2,175.980	0.987990
CN03	5.774238	1,825.091	1,857.022	0.990863
CN04	5.781661	1,894.323	1,932.649	0.990009
CN05	5.629790	2,020.282	2,073.000	0.988585
CN06	5.831293	2,180.018	2,211.477	0.987938
SF01	3.821835	1,301.581	1,339.524	0.992131
SF02	2.530843	1,060.410	1,060.000	0.993296
SF03	2.966455	965.6678	960.7213	0.994202
SF04	3.040859	1,061.814	1,076.025	0.993762
SF05	3.459051	1,221.529	1,261.000	0.993244
SF06	2.630863	1,066.978	1,069.048	0.993374
SF07	4.690538	1,439.223	1,447.628	0.992131
SF08	4.759374	1,428.891	1,445.189	0.992131
SF09	3.687505	1,100.771	1,127.982	0.993684
SF10	2.456487	849.9415	816.6935	0.994901
SF11	3.290117	1,139.317	1,130.511	0.993322
SF12	3.252698	460.3691	441.3860	0.997463
SF13	3.505656	1,173.848	1,203.848	0.992960
SF14	2.410852	818.7996	813.6535	0.994461
SF15	3.905919	1,182.603	1,171.882	0.993503

**Figure 5:**
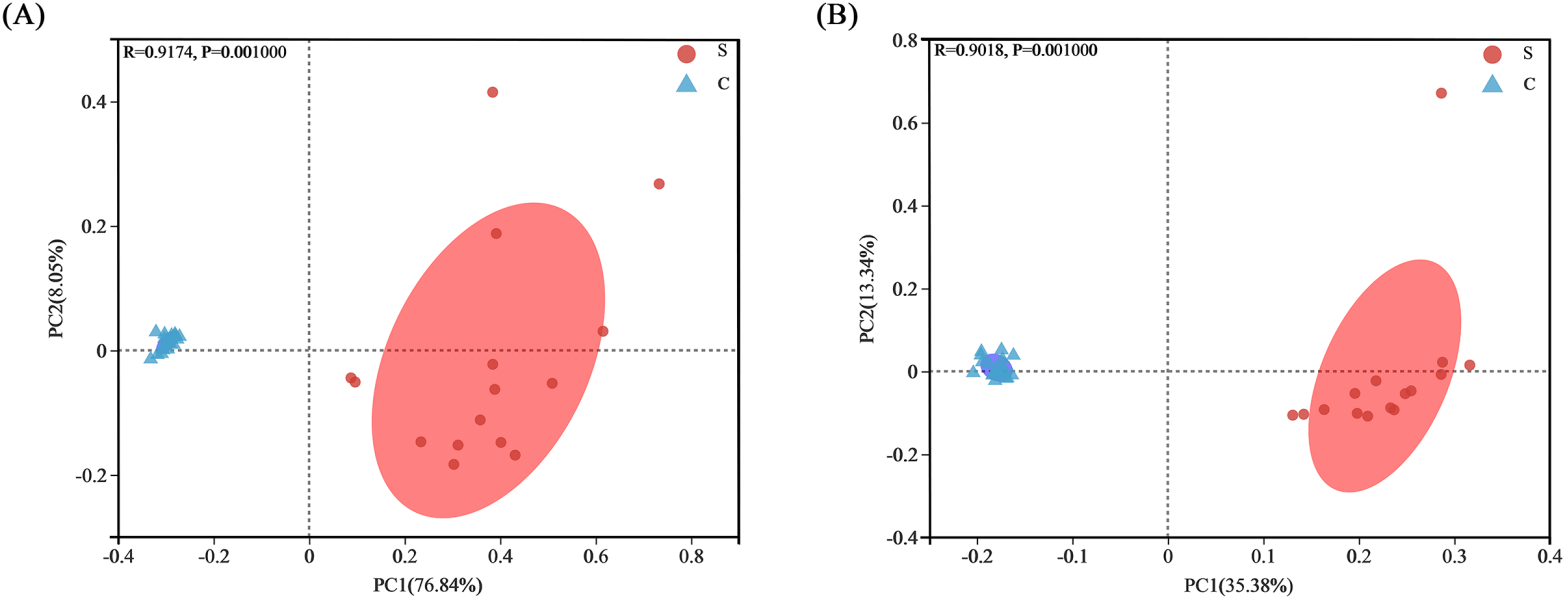
Analysis of PCoA plots of gut microbiome from Père David’s deer. (A) PCoA plots based on weighted UniFrac distances. (B) PCoA plots based on unweighted UniFrac distances.

Bacterial community clusters in all samples were visualized by PCoA plots under both weighted and unweighted UniFrac metrics, with each symbol representing an intestinal flora on the PCoA plot. Most samples within the S group and C group were close together and highly aggregated, so they could be distinguished from samples in the other group. The bacterial communities of the S group were separated from those of the C group along main axis 1 (PC1) using weighted UniFrac distances, with the greatest amount of variation (76.84%). When using the unweighted UniFrac distance, the amount of variation reached 35.38% (Fig. 5). This result indicated a high similarity in gut microbiota composition within each group at the OTU level, but differing significantly between the S group and C group.

### Differences across age and sex in individuals in the captivity group

**Figure 6:**
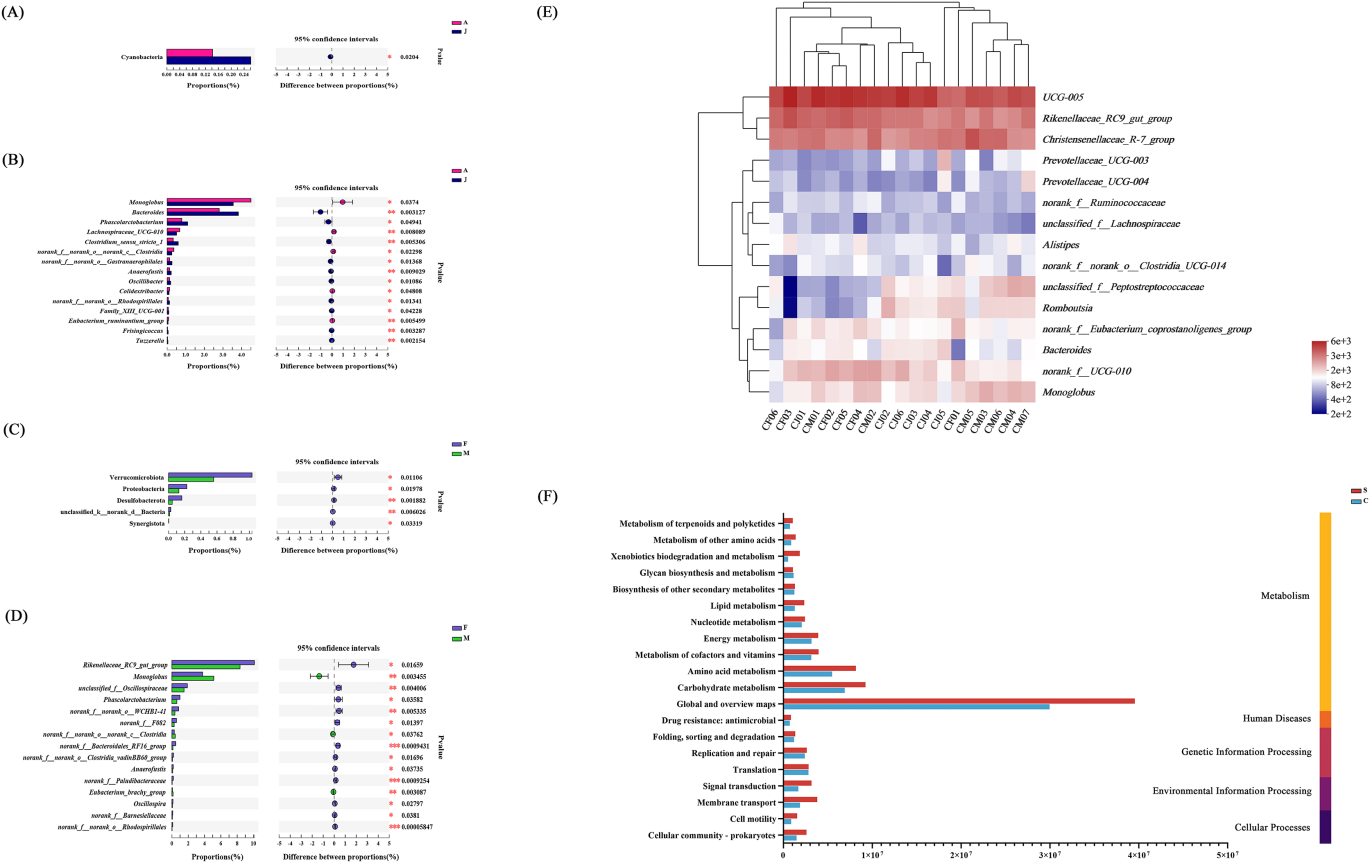
Analysis of intestinal flora in individuals of different ages and genders. Differential analysis of dominant bacterial phyla (A) and genera (B) between adult and juvenile groups; Differential analysis of dominant bacterial phyla (C) and genera (D) between male and female groups; * *P* < 0.05, ** *P* < 0.01, *** *P* < 0.001. (E) Clustering analysis of the evolution of gut microbiota in the C group based on the Bray–Curtis distances generated by mothur; (F) gut microbial function prediction of total individuals based on KEGG databases.

Compared with the gut microbiota from adult individuals, the abundance of Cyanobacteria at the phylum level and *Bacteroides*, *Phascolarctobacterium*, *Clostridium_sensu_stricto_1* at the genus level were significantly higher in the juvenile individuals compared with adult individuals (Fig. 6A). However, the relative abundance of *Monoglobus*, *Lachnospiraaceae_UGG-010*, and *norank_f_norank_o_norank_c_Clostridia* in adult individuals were significantly higher than juveniles (Fig. 6B).

We analyzed the effect of sex on the composition of gut microbiota, and found that at the phylum level, the abundances of Verrucomicrobiota, Proteobateria, and Desulfobaterota were higher in female individuals (Fig. 6C). At the genus level, *Rikenellaceae_RC9_gut_group*, *unclassified_f_Oscillospiraceae*, *Phascolarctobacterium*, and *norank_o_WCHB1-41* were more abundant in female than male individuals, whereas the males had a significantly higher relative abundance of *Monoglobus* and *norank_f_norank_o_norank_c_Clostridia* compared with females (Fig. 6D). The community heat map analysis between individuals of different ages and sex at the genus level is shown in Fig. 6E.

### Function prediction of gut microbiota communities

Based on high-throughput sequencing, a total of 46 KEGG pathways were mapped and then divided into secondary KEGG pathways (Fig. S3). The gut microbial function was predicted based on the Clusters of Orthologous Genes (COG) database (Fig. S4). The secondary KEGG pathways related to gut microbiota included metabolism, genetic information processing, and environmental information processing, which demonstrated that differences in gut microbiota had remarkable influence on the metabolism of *E. davidianus* (Fig. 6).

## Discussion

In this study, Firmicutes and Bacteroidota were the dominant phyla in Père David’s deer from different living conditions, consistent with previous conclusions in this endangered species (Sun et al., 2019). Previous studies have shown that the gut microbiota of ruminants *e.g*., white-lipped deer (*Cervus albirostris*) (Li et al., 2022; You et al., 2022), forest musks (*Moschus berezovskii*) (Zhao et al., 2021), alpine musks (*Moschus sifanicus*) (Jiang et al., 2021), and blue sheep (*Pseudois nayaur*) (Zhu et al., 2020), were predominantly made up by Firmicutes and Bacteroidota. This was mainly because ruminants are herbivores that need microorganisms to help them digest and absorb nutrients from plants (Zhu et al., 2020). The bacteria of Firmicutes could encode enzymes that promote energy metabolism by utilizing a variety of substances (Kaakoush, 2015), and many have the function of degrading carbohydrates including cellulose and starch, as well as fat (Jiang et al., 2021). For example, *UCG-005* is a cellulose-degrading bacterium (Li et al., 2020), and *norank_f_UCG-010* is important for energy uptake in ruminants (Guan et al., 2017; Hassan et al., 2021; Zhang et al., 2022). They were the dominant genera in both semi-free-ranging and captive Père David’s deer.

The dynamics of intestinal flora is an important mechanism by which the host adapts to environmental changes (Moeller & Sanders, 2020). One of the main factors affecting the intestinal flora is diet (Fernando et al., 2010; Zhen et al., 2022). The variety in microbial community composition caused by differences in forage has been demonstrated (Henderson et al., 2015). *Christensenellaceae_R-7_group* belonging to Firmicutes, which was enriched in captive individuals, was predominantly associated with carbohydrate metabolism and energy metabolism. The result was consistent with previous findings in the same species by Wang et al. (2019). *Monoglobus* and *norank_f_Eubacterium_coprostanoligenes_group*, which also belong to Firmicutes, are intestinal microorganisms that specifically degrade pectinand contribute to the absorption and utilization of fat, respectively (Kim et al., 2019; Wei et al., 2021). The forage for captive Père David’s deer in Tianjin Zoo mainly consisted of wheat bran, corn, soybean, and sorghum which are wealthy in starch, protein, fat, and fiber (Maloiy et al., 1970). Thus, this diet structure led to the enrichment of Bacteroidota (Zhao et al., 2019), which are involved in the degradation of macromolecular compounds such as proteins and carbohydrates (Jami, White & Mizrahi, 2014; Hu et al., 2017). For example, *Rikenellaceae_RC9_gut_group* the dominant genera in captive individuals, is beneficial bacteria in the gut which promote host health and degradation of structural carbohydrates including lignin and cellulose (Zened et al., 2013; Qin et al., 2022).

Previous work had shown that the ratio of Firmicutes and Bacteroidota (F/B) was often associated with the digestion and absorption of carbohydrate-rich foods (Turnbaugh et al., 2006), increasing the ability to metabolize fat (Backhed et al., 2005). This ratio was higher in the S group compared with the C group, since the main foods were grasses, such as gramineous plants which have poor survival during winter for the semi-free-ranging individuals. A higher ratio usually implies a greater ability to absorb nutrients (Mariat et al., 2009; Jami, White & Mizrahi, 2014). Therefore, we presume that semi-free-ranging individuals need to improve the efficiency of energy extraction from ‘poor quality food’ (Fernando et al., 2010) in order to adapt to the harsh natural conditions (Zhao et al., 2019). The genus *Paenisporosarcina* survives in cold regions and is an important plant rhizosphere microorganism with anti-freeze functions (Zheng et al., 2018). Accordingly, we speculated that its enrichment within the intestine may come from natural foods eaten in the winter. In our study, the abundances of Proteobacteria and Actinobacteriota in the S group were significantly higher than the C group. The genera *Pseudomonas* and *Acinetobacter*, belonging to Proteobacteria, were enriched in the S group. They are conditionally pathogenic bacteria, and can cause an inflammatory response in the organism (Von Klitzing et al., 2017). It has been shown that the aggregation of Proteobacteria can be used as an indicator of dysbiosis (Shin, Whon & Bae, 2015; Zhao et al., 2019), and the abundance of *Acinetobacter* in forest musk individuals with pneumonia was significantly higher than that in healthy individuals (Zhao et al., 2021). The diet structure of Père David’s deer in a semi-free-ranging state was unstable since additional forage was provided due to limited plant resources in winter (Zhao et al., 2019; Zhen et al., 2022). The results showed that alpha diversity was significantly higher in the C group than the S group (*P* < 0.01), which was similar to many other studies on Cervidae species (Li et al., 2020; Minich et al., 2021). Captive feeding may increase alpha diversity due to the adequate high-fiber food provided by zoos in the winter (Guan et al., 2017).

In our study, we found that individuals of different ages and gender did not differ significantly in alpha diversity of gut microbiota, but there were significant differences in flora composition, which was consistent with the research on forest musks (Zhao et al., 2019). It has been experimentally demonstrated that there are changes in total food intake and energy acquisition in animals at different ages (Passadore et al., 2004), leading to variation in intestinal flora (Jiang et al., 2020). The composition of intestinal flora shows significant differences in many ruminants before and after weaning (Jami et al., 2013; Li et al., 2020). In our study, the abundance of *Bacteroides*, which has the function of digesting fat and protein, was significantly higher in the intestine of juvenile individuals than in adult individuals. We hypothesized that it facilitated the absorption of nutrients required during development in juvenile individuals. Since forage was the main food for adult individuals, the contents of the genus *Monoglobus*, which can degrade the pectin component of plant cell walls, was significantly richer in the gut of adult individuals than juveniles (Jewell et al., 2015). The enrichment of *Lachnospiraceae_UGG-010* in adult individuals may be positively correlated with feed utilization. In addition, the results showed that the genus *Phascolarctobacterium* was also enriched in the gut of juvenile individuals. Since the decrease of *Phascolarctobacterium* could lead to an imbalance in human host immune homeostasis (Chen et al., 2021), we speculated that *Phascolarctobacterium* could protect young individuals from disease (Oikonomou et al., 2013), and thus the composition of gut microbiota in deer was strongly related to the immune function of the host (Moeller & Sanders, 2020).

Numerous studies on animals and humans have shown that gender affects the structure of the intestine flora (Kim et al., 2020; Minich et al., 2021). In our study, only captive individuals were analyzed to find the influence of gender on gut microbiota. It has been shown that adult females invest larger amounts of energy reserves and consume food to produce offspring (Moyes et al., 2006). In this study, the abundances of *Rikenellaceae_RC9_gut_group* and *Phascolarctobacterium* were significantly higher in females than in males, promoting host nutrient absorption and health as mentioned previously (Chen et al., 2021; Qin et al., 2022). Furthermore, the results showed that the abundances of both the phylum Verrucomicrobiota and the genus *norank_o_WCHB1-41* belonging to this phylum were significantly higher in females than in males. Since Verrucomicrobiota can degrade many complex polysaccharides (Sichert et al., 2020), *norank_o_WCHB1-41* might be the genus that plays a key role in this phylum (Hassan et al., 2021). At present, the influence of intestinal microorganisms on Père David’s deer is based on our analysis and previous reports, and further experiments are needed to verify this potential link.

## Conclusions

Reintroduction is an important behavioral measure for endangered species conservation, and the process of adaptation to the environment during species reintroduction needs scientific monitoring. The detection of intestinal microorganisms provides a new idea for the reintroduction and protection of wildlife. This study is the first report to compare the differences in gut microbiota composition of Père David’s deer in different habitats from the warm temperate zone. It was found that there are significant differences in gut microbiota composition and diversity between the captive group and semi-free-ranging group, and that age and gender also affect the composition of the gut microbiota under the same feeding condition. This research provides comparative information of gut microbiota from Père David’s deer in northern China, which will be useful to further understand how living conditions affect gut microbiota of this endangered species and provide a scientific reference for successful introductions.

## Supplemental Information

10.7717/peerj.14897/supp-1 Supplemental Information 1 Tianjin Zoo captive Père David’s deer pellet feed composition.

10.7717/peerj.14897/supp-2 Supplemental Information 2 The total number of raw reads, base pairs, the mean length of the reads.

10.7717/peerj.14897/supp-3 Supplemental Information 3 Mean relative abundance of the 10 most abundant phyla in Tianjin Zoo and Qilihai Wetland.

10.7717/peerj.14897/supp-4 Supplemental Information 4 Mean relative abundance of the 10 most abundant gerna in Tianjin Zoo and Qilihai Wetland.

10.7717/peerj.14897/supp-5 Supplemental Information 5 The Rarefaction curve and OTUs of Père David’s deer from two groups. Rarefaction curve indicating a adequate number of OTUs be detected from C group (A) and S group (B); venn diagram showing the unique and shared gut bacterial OTUs between two groups (C)

10.7717/peerj.14897/supp-6 Supplemental Information 6 Histogram showing the T-test results of Alpha diversity. The index curves of ACE (A),Shannon (B) and Chao1 (C) between C group and S group

10.7717/peerj.14897/supp-7 Supplemental Information 7 Gut microbial function prediction of total individuals based on KEGG databases.

10.7717/peerj.14897/supp-8 Supplemental Information 8 Gut microbial function prediction of total individuals based on COG databases.

## Additional Information and Declarations

### Data Availability

The following information was supplied regarding data availability: All raw sequence reads in this study are available at Sequence Read Archive (SRA-NCBI): PRJNA877437; SRR21465239 to SRR21465272.
