# Supplementary material for: How do living conditions affect the gut microbiota of endangered Père David’s deer (Elaphurus davidianus)? Initial findings from the warm temperate zone
Source: PeerJ. 2023 Feb 24;11:e14897. doi: 10.7717/peerj.14897 (PMC9969852; doi:10.7717/peerj.14897)
Supplement: Supplemental Information 1 [file peerj-11-14897-s001.docx]

Supplementary Table1. Tianjin Zoo captive Père David's deer pellet feed composition

| **Nutritional Composition** | **Content（%）** |
| --- | --- |
| Cornmeal | 40 |
| Sorghum noodles | 10 |
| Wheat bran | 12 |
| Bone meal | 2 |
| Salt | 1 |
| Soybean meal | 15 |
| Alfalfa powder | 18 |
| Fish Meal | 2 |
