# Supplementary material for: How do living conditions affect the gut microbiota of endangered Père David’s deer (Elaphurus davidianus)? Initial findings from the warm temperate zone
Source: PeerJ. 2023 Feb 24;11:e14897. doi: 10.7717/peerj.14897 (PMC9969852; doi:10.7717/peerj.14897)
Supplement: Supplemental Information 2 [file peerj-11-14897-s002.docx]

Supplementary Table2.The total number of raw reads, base pairs, the mean length of the reads.

| **Sample** | **Seq_num** | **Base_num** | **Mean_length** | **Min_length** | **Max_length** |
| --- | --- | --- | --- | --- | --- |
| CF01 | 224397 | 92278536 | 411.2289 | 223 | 452 |
| CF02 | 278338 | 1.15E+08 | 412.5381 | 277 | 433 |
| CF03 | 232925 | 96158854 | 412.8318 | 260 | 431 |
| CF04 | 280489 | 1.16E+08 | 411.9304 | 378 | 442 |
| CF05 | 267042 | 1.1E+08 | 411.9691 | 317 | 431 |
| CF06 | 52684 | 21706249 | 412.0084 | 234 | 431 |
| CJ01 | 248379 | 1.02E+08 | 411.9311 | 219 | 432 |
| CJ02 | 306943 | 1.26E+08 | 411.4846 | 320 | 441 |
| CJ03 | 46730 | 19235749 | 411.636 | 249 | 431 |
| CJ04 | 46848 | 19265980 | 411.2445 | 249 | 434 |
| CJ05 | 46544 | 19217137 | 412.8811 | 249 | 450 |
| CJ06 | 267268 | 1.1E+08 | 411.4491 | 204 | 431 |
| CM01 | 286304 | 1.18E+08 | 411.664 | 204 | 436 |
| CM02 | 275676 | 1.13E+08 | 411.0578 | 219 | 451 |
| CM03 | 43036 | 17686457 | 410.9689 | 249 | 431 |
| CM04 | 43392 | 17846088 | 411.276 | 292 | 431 |
| CM05 | 50242 | 20689093 | 411.7888 | 249 | 432 |
| CM06 | 46625 | 19196235 | 411.7155 | 252 | 431 |
| CM07 | 48165 | 19846837 | 412.0593 | 249 | 431 |
| SF01 | 270283 | 1.14E+08 | 421.1406 | 302 | 448 |
| SF02 | 230382 | 97706922 | 424.1083 | 204 | 434 |
| SF03 | 245811 | 1.04E+08 | 423.0437 | 235 | 452 |
| SF04 | 281643 | 1.18E+08 | 418.8463 | 277 | 452 |
| SF05 | 259346 | 1.09E+08 | 420.6556 | 291 | 450 |
| SF06 | 263236 | 1.11E+08 | 420.9288 | 249 | 431 |
| SF07 | 272292 | 1.12E+08 | 412.9615 | 258 | 449 |
| SF08 | 276473 | 1.14E+08 | 412.6238 | 326 | 451 |
| SF09 | 249059 | 1.05E+08 | 420.2791 | 337 | 446 |
| SF10 | 255500 | 1.09E+08 | 425.0845 | 304 | 432 |
| SF11 | 232233 | 97703170 | 420.7118 | 219 | 452 |
| SF12 | 271172 | 1.12E+08 | 411.7861 | 232 | 431 |
| SF13 | 253238 | 1.06E+08 | 420.153 | 328 | 435 |
| SF14 | 221532 | 94533750 | 426.7273 | 328 | 449 |
| SF15 | 248546 | 1.04E+08 | 418.2944 | 229 | 431 |
