# Supplementary material for: How do living conditions affect the gut microbiota of endangered Père David’s deer (Elaphurus davidianus)? Initial findings from the warm temperate zone
Source: PeerJ. 2023 Feb 24;11:e14897. doi: 10.7717/peerj.14897 (PMC9969852; doi:10.7717/peerj.14897)
Supplement: Supplemental Information 3 [file peerj-11-14897-s003.docx]

Supplementary Table3. Mean relative abundance of the 10 most abundant phyla in Tianjin Zoo and Qilihai Wetland

| **Sample group** | **Top ten abundant phyla (%)** |
| --- | --- |
| Tianjin Zoo (Z group) | Firmicutes(67.89) |
|  | Bacteroidota(28.43) |
|  | Actinobacteriota(0.85) |
|  | Verrucomicrobiota(0.78) |
|  | Patescibacteria(0.71) |
|  | Spirochaetota(0.69) |
|  | Proteobacteria(0.18) |
|  | Cyanobacteria(0.18) |
|  | Fibrobacterota(0.12) |
|  | Desulfobacterota(0.11) |
| Qilihai Wetland (S group) | Firmicutes(68.02) |
|  | Proteobacteria(17.74) |
|  | Actinobacteriota(10.09) |
|  | Bacteroidota(3.47) |
|  | Cyanobacteria(0.27) |
|  | Patescibacteria(0.20) |
|  | Verrucomicrobiota(0.09) |
|  | Fibrobacterota(0.07) |
|  | Bacteria(0.01) |
|  | Spirochaetota(0.01) |
