# Supplementary material for: How do living conditions affect the gut microbiota of endangered Père David’s deer (Elaphurus davidianus)? Initial findings from the warm temperate zone
Source: PeerJ. 2023 Feb 24;11:e14897. doi: 10.7717/peerj.14897 (PMC9969852; doi:10.7717/peerj.14897)
Supplement: Supplemental Information 4 [file peerj-11-14897-s004.docx]

Supplementary Table4. Mean relative abundance of the 10 most abundant gerna in Tianjin Zoo and Qilihai Wetland

| **Sample group** | **Top ten abundant gerna (%)** |
| --- | --- |
| Tianjin Zoo (Z group) | *UCG-005*(13.05) |
|  | *Rikenellaceae_RC9_gut_group*(8.94) |
|  | *Christensenellaceae_R-7_group*(8.37) |
|  | *norank_f__UCG-010*(4.64) |
|  | *Monoglobus*(4.20) |
|  | *norank_f__Eubacterium_coprostanoligenes_group*(3.50) |
|  | *unclassified_f__Peptostreptococcaceae*(3.22) |
|  | *Romboutsia*(3.20) |
|  | *Bacteroides*(3.12) |
|  | *Alistipes*(2.69) |
| Qilihai Wetland (S group) | *Psychrobacillus*(26.53) |
|  | *Pseudomonas*(11.33) |
|  | *UCG-005*(7.98) |
|  | *Arthrobacter*(6.22) |
|  | *Paenisporosarcina*(4.58) |
|  | *Acinetobacter*(3.71) |
|  | *Sporosarcina*(3.42) |
|  | *norank_f__UCG-010*(3.04) |
|  | *norank_f__Eubacterium_coprostanoligenes_group*(2.80) |
|  | *Christensenellaceae_R-7_group*(2.77) |
