# Supplementary figures and images for: How do living conditions affect the gut microbiota of endangered Père David’s deer (Elaphurus davidianus)? Initial findings from the warm temperate zone
Source: PeerJ. 2023 Feb 24;11:e14897. doi: 10.7717/peerj.14897 (PMC9969852; doi:10.7717/peerj.14897)

(A)

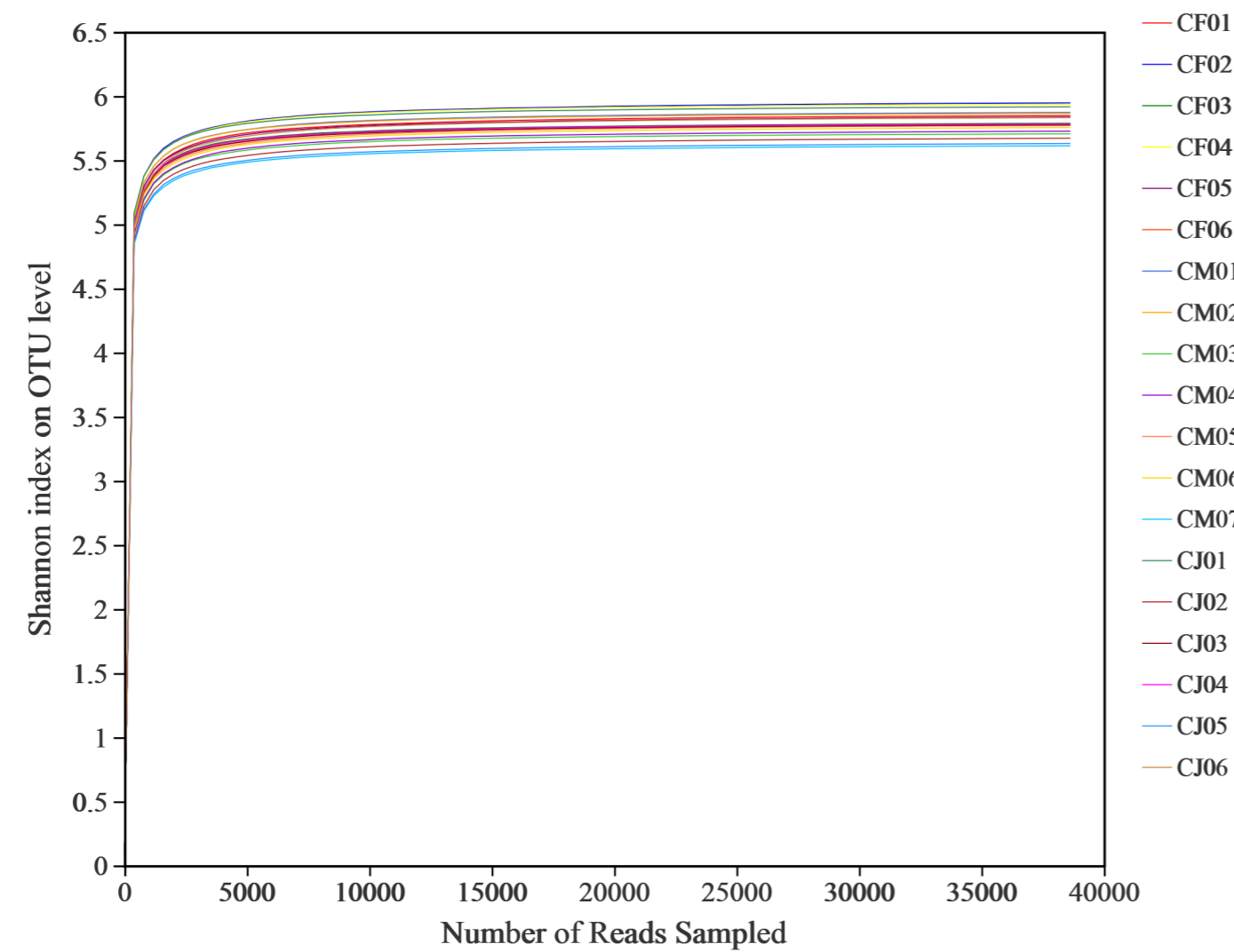

(B)

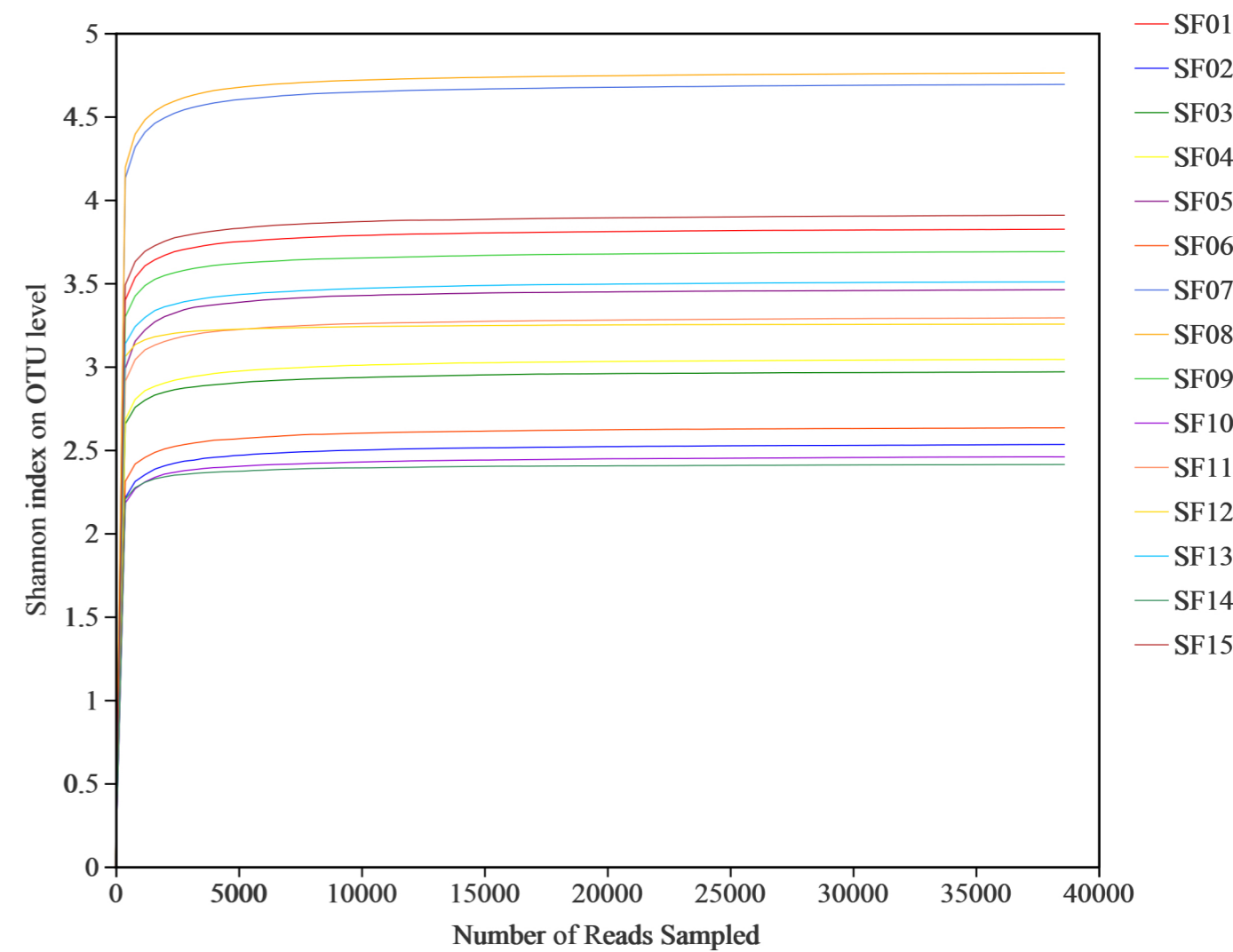

(C)

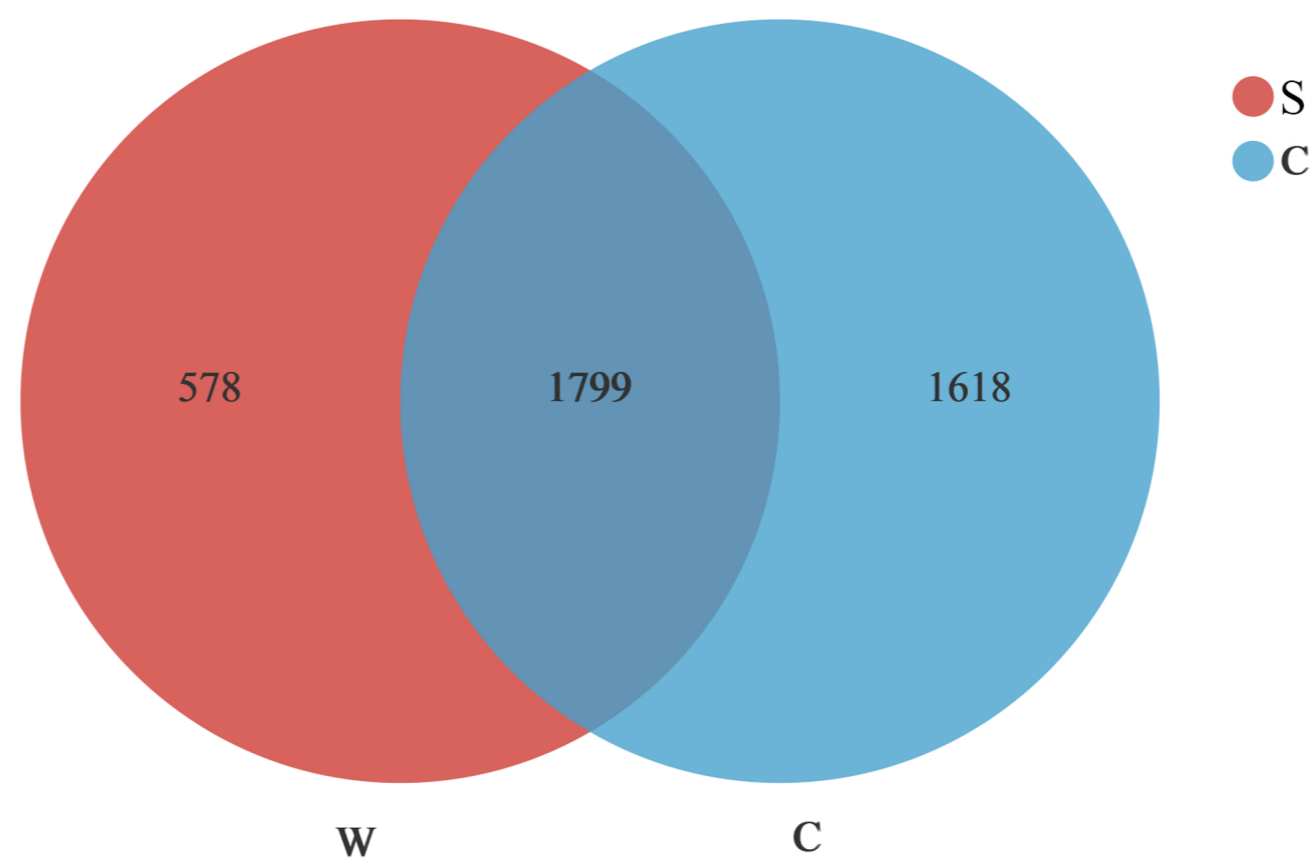

Supplement: Supplemental Information 5 — Rarefaction curve indicating a adequate number of OTUs be detected from C group (A) and S group (B); venn diagram showing the unique and shared gut bacterial OTUs between two groups (C) [file peerj-11-14897-s005.pdf]

(A)

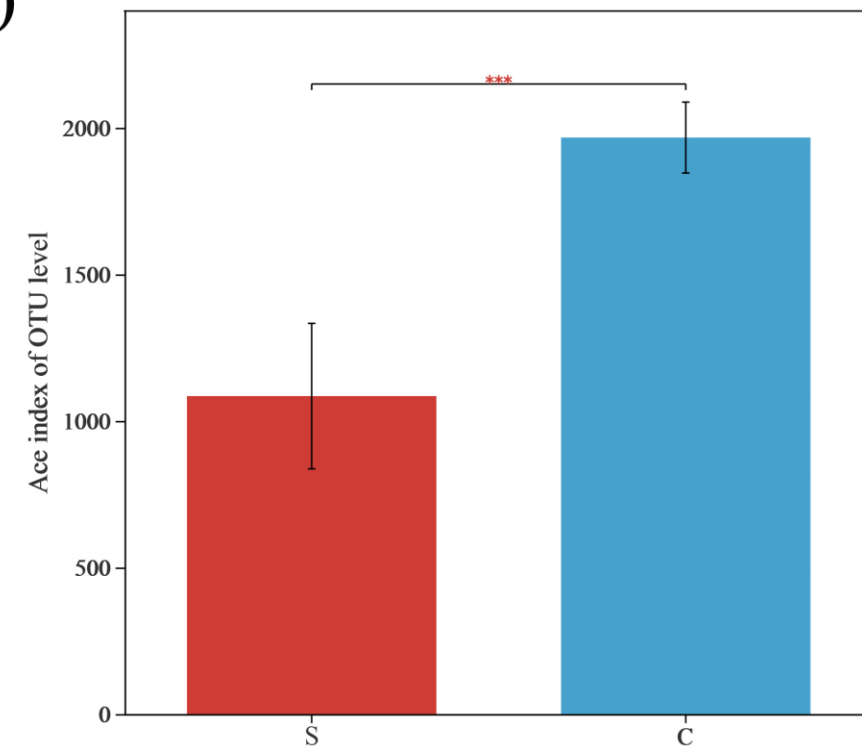

(B)

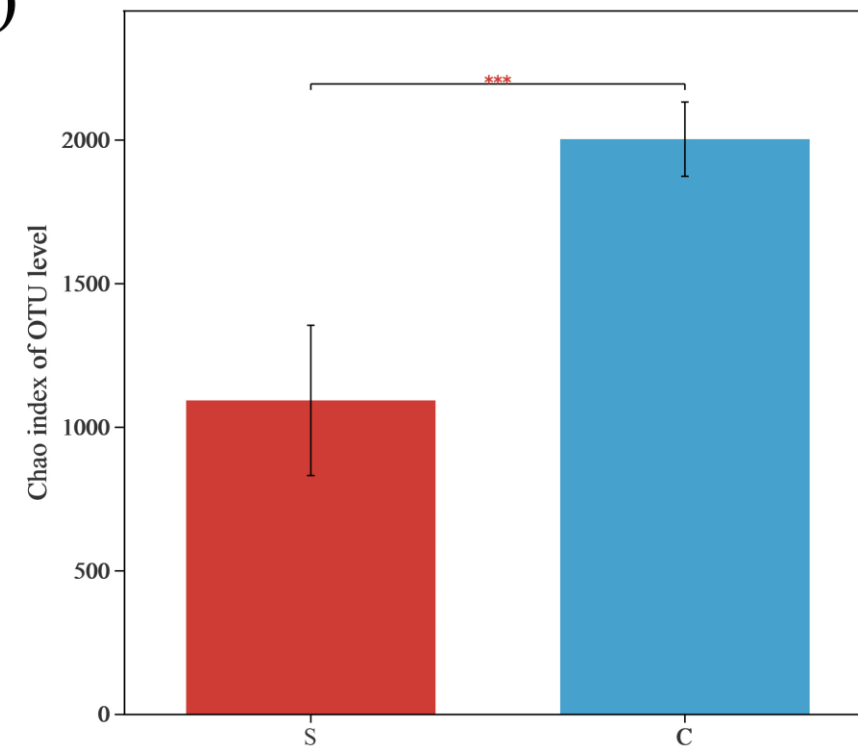

(C)

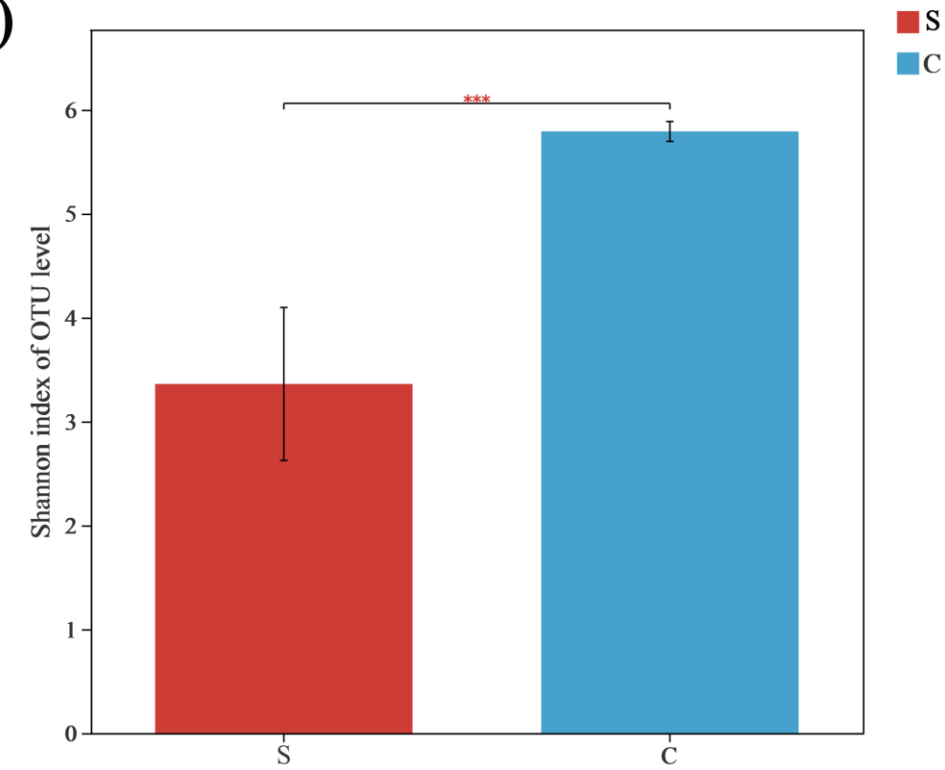

Supplement: Supplemental Information 6 — The index curves of ACE (A),Shannon (B) and Chao1 (C) between C group and S group [file peerj-11-14897-s006.pdf]

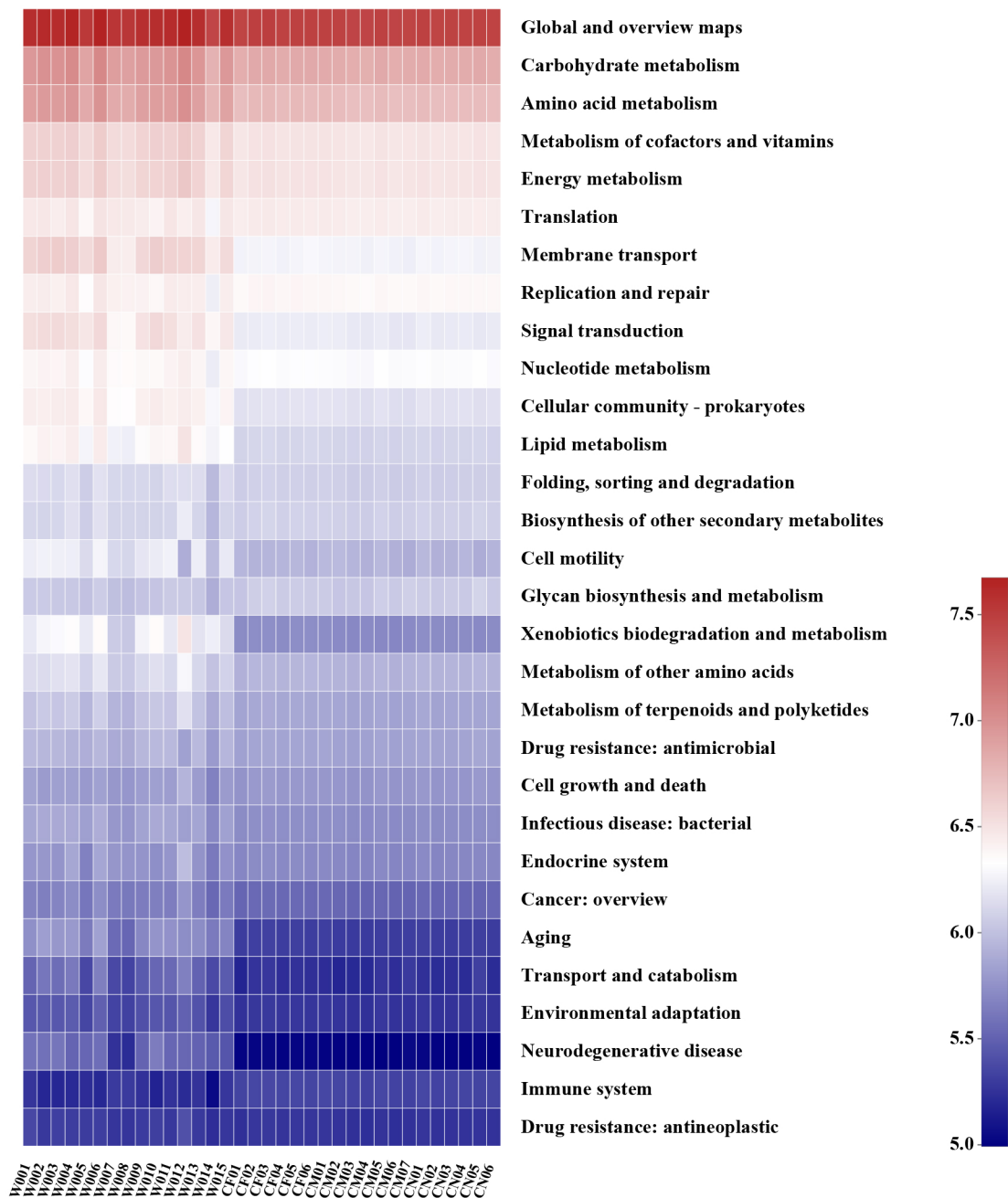

Supplement: Supplemental Information 7 [file peerj-11-14897-s007.pdf]

# COG function classification

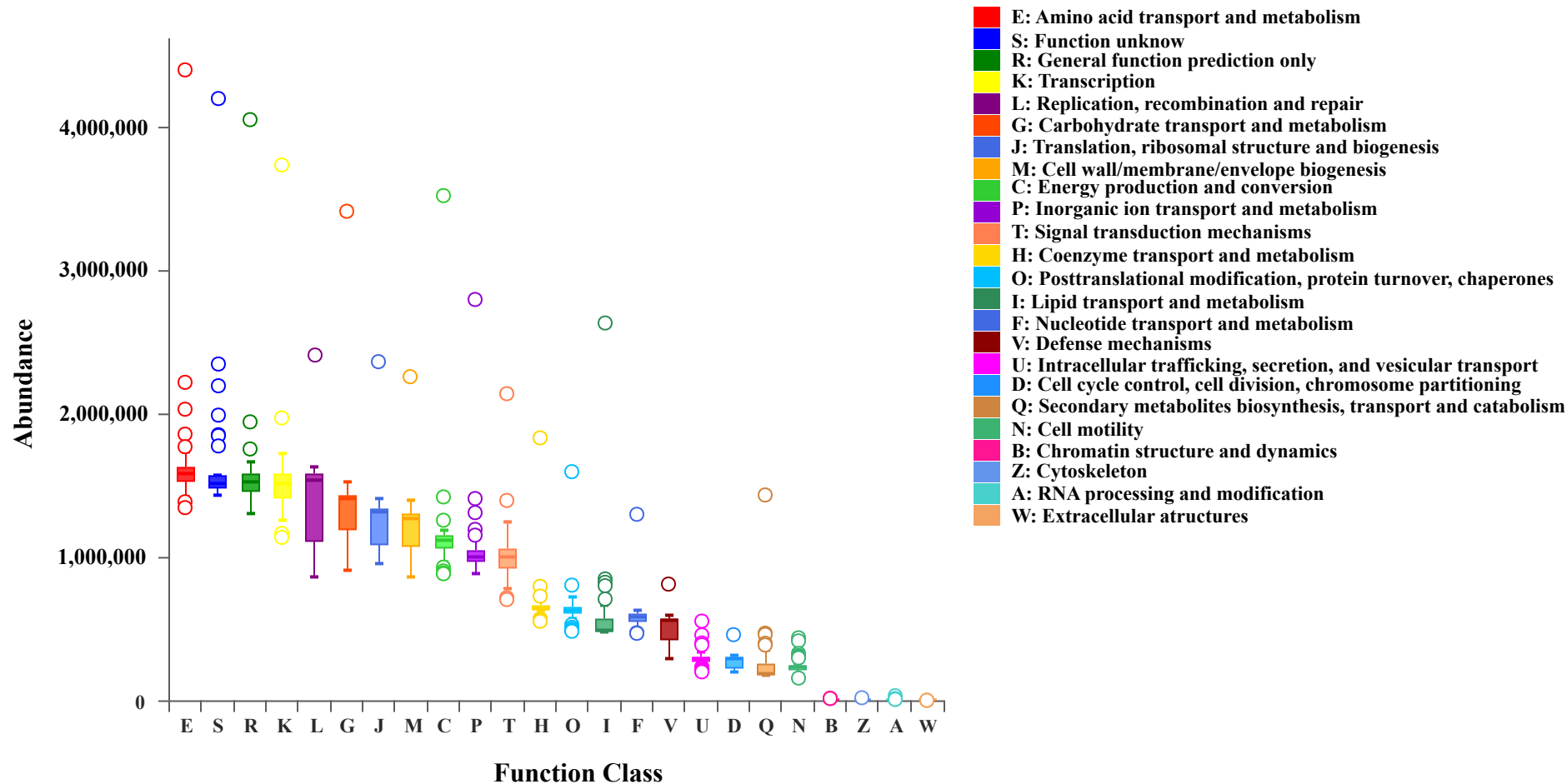

Supplement: Supplemental Information 8 [file peerj-11-14897-s008.pdf]
